# Interfacial Polarization of Thin Alq_3_, Gaq_3_, and Erq_3_ Films on GaN(0001)

**DOI:** 10.3390/ma15051671

**Published:** 2022-02-23

**Authors:** Miłosz Grodzicki, Jakub Sito, Rafał Lewandków, Piotr Mazur, Antoni Ciszewski

**Affiliations:** Institute of Experimental Physics, University of Wroclaw, Pl. M. Borna 9, 50-204 Wroclaw, Poland; jakub.sito@uwr.edu.pl (J.S.); rafal.lewandkow@uwr.edu.pl (R.L.); piotr.mazur@uwr.edu.pl (P.M.); antoni.ciszewski@uwr.edu.pl (A.C.)

**Keywords:** Mq_3_, GaN, polarization, organic layers, electronic structure

## Abstract

This report presents results of research on electronic structure of three interfaces composed of organic layers of Alq_3_, Gaq_3_, or Erq_3_ deposited on GaN semiconductor. The formation of the interfaces and their characterization have been performed in situ under ultrahigh vacuum conditions. Thin layers have been vapor-deposited onto p-type GaN(0001) surfaces. Ultraviolet photoelectron spectroscopy (UPS) assisted by X-ray photoelectron spectroscopy (XPS) has been employed to construct the band energy diagrams of the substrate and interfaces. The highest occupied molecular orbitals (HOMOs) are found to be at 1.2, 1.7, and 2.2 eV for Alq_3_, Gaq_3_, and Erq_3_ layers, respectively. Alq_3_ layer does not change the position of the vacuum level of the substrate, in contrast to the other layers, which lower it by 0.8 eV (Gaq_3_) and 1.3 eV (Erq_3_). Interface dipoles at the phase boundaries are found to be −0.2, −0.9, −1.2 eV, respectively, for Alq_3_, Gaq_3_, Erq_3_ layers on GaN(0001) surfaces.

## 1. Introduction

Gallium nitride (GaN) is a very attractive semiconductor for applications in optoelectronics and photovoltaics. It is also used for creating high-power and -frequency devices [1,2]. GaN is one of the most commonly used materials for fabricating devices in the mentioned electronic areas. This occurs due to its good physicochemical properties, such as a direct and wide band gap, high thermal conductivity, and thermal stability. The III-nitrides semiconductors also have a potential in creating three-dimensional hybrid organic/inorganic electronic devices, such as organic light-emitting diodes (LEDs), organic field-effect transistors (OFETs), or biosensors [3,4]. The use of organic semiconductors from the Mq_3_ chelate group (M—trivalent metal, q—8-hydroxyquinoline) as an active element of electronic devices has been known for years. It began with Tang’s report, which showed potential possibilities of Alq_3_ [5]. In recent times, Mq_3_ molecules seem to have been great candidates for applying to a light-emitting and/or electron-transporting material in hybrid technology. This is because of the merging of the high charge carrier mobility and efficient charge injection of inorganic semiconductors with the strong light-matter coupling and large chemical composition diversity of organic semiconductors [6,7,8]. Mq_3_ complexes are promising materials for sensor and biosensor applications, the main reason is their ability to interact with a wide range of analytes, such as p-nitroaniline, NO_2_, ethanol, and methanol [9]. Another relevant application is the incorporation of Mq_3_ compounds to improve OLED’s device design [10]. Lately, Erq^3^ was used to exceed the limitation of the exaction production efficiency of NIR OLED’s over the theoretical limit of 100%, which can lead to light sources exceeding the intensity of the OLEDs produced in current technology [11]. The use of Gaq_3_ allowed for increased efficiency of the solar cells [12] and OLED’s [13] and application of Alq_3_ as an acceptor material in the UV-photodetector [14]. With the advancement of technology and making it possible to produce layers with better properties, there has been rediscovered interest in the organic/inorganic hybrid structures in search of new functionalities in various fields of study. Organic materials are projected to enter the GaN-based hybrid device field [15]. The interfacial polarization of inorganic-organic heterojunction is important because it brings steep shifts in electronic band structure across interfaces and thus effectively tunes charge carrier transport. One of the possible ways to modify charge injection behavior in inorganic-organic heterojunction devices is to make use of interfacial polarization caused by the partial alignment of the permanent dipole moments of polar organic molecules [16,17]. Mq_3_ molecules have a large electric dipole moment (~4 D) [18,19]. Mq_3_ molecules have been widely studied for their potential applications, inter alia, in organic solar cells, light emission diodes, and data storage and communication devices [20,21,22,23]. Regarding this, the molecule/GaN systems are attractive for both industry and academic research. H. Kim et al. in work [24] proposed using an Alq_3_ layer in GaN-based heterostructures. Apart from Alq_3_, the Gaq_3_, and Erq_3_ appear to be new candidates for applying to such structures. An important issue in the context of such systems is the electronic structure of the interface, in particular the interfacial polarization or the position of the highest occupied molecular orbital (HOMO) level of molecules relative to the valence band maximum (VBM) of the substrate. So far, this information has been omitted in reports on Mq_3_ films on GaN(0001) surface. The interfacial polarization has an impact on the band offset at the interface and this, in turn, has a bearing on the current–voltage characteristics of inorganic-organic devices. The tuning effect of central atom M in Mq_3_ molecules on the band offset is of application importance.

This report presents a basic study of Alq_3_, Gaq_3_, and Erq_3_ layers on GaN(0001) surfaces. The research focused on the electronic properties of the resulting interfaces and was carried out using ultraviolet photoelectron spectroscopy (UPS) assisted by X-ray photoelectron spectroscopy (XPS). The main goal was to check the capability of used Mq_3_ to tune the position of HOMO and vacuum levels for the systems formed with p-GaN(0001) surfaces.

## 2. Materials and Methods

In this experiment, gallium nitride p-type, (0001)-oriented, on which Mq_3_ films were deposited, was used as a substrate. Mg dopant concentration was ∼1 × 10^18^ cm^−3^. The GaN(0001) samples around 5 × 10 mm^2^ in size were cut from one wafer grown by metalorganic chemical vapor deposition. Initial bare surfaces with a trace of residual oxygen were achieved by degassing GaN samples mounted on Mo plates. The samples were thermally annealed up to 500 °C. A radiation heater in an ultrahigh vacuum (UHV) chamber with a base pressure lower than 1 × 10^−^^10^ Torr was utilized. The temperature was monitored by a pyrometer. The three Mq_3_/GaN(0001) systems were grown in situ by evaporation of molecules from quartz crucibles heated with thermal radiation. The calibration of the sources was done by means of XPS [25,26,27,28]. The 1.5 nm attenuation length of electrons with a kinetic energy of ∼370 eV in organic layers was used to evaluate the film thicknesses and thus growth rates. The parameter was calculated based on NIST Standard Reference Database [29]. The films were deposited step by step up to 15 nm. Adsorbate dosages were established from the evaporation time after the source temperature had stabilized. A surface-analysis system (Specs) was employed for in situ characterization. The main technique used was UPS, and the second was XPS. The XPS data collected in this experiment suggest the growth mode of the Mq_3_ layers on the substrate, although the study has not focused on determining it. The decrease in the intensity of the Ga 2p core-level line with the increase in the Mq_3_ thickness shows that the data are closest to the theoretical prediction of the Volmer–Weber growth mode. It indicates 3D growth mode for all three Mq_3_ films. The photoemission experiments were carried out using a hemispherical electron energy analyzer (Phoibos 100) and a UPS source with He I (21.2 eV) excitation line, and two X-ray non-monochromatic radiation sources, i.e., Mg Kα (1253.6 eV) and Al Kα (1486.6 eV). Photoelectrons were collected in the CAE mode with a pass energy of 2 or 10 eV and a step size of 0.025 or 0.1 eV, respectively, for UPS and XPS measurements. During measurement, the optical axis of the analyzer entrance was normal to the substrate surface. Binding energy values refer to the Fermi level (*E*_F_) of the electron analyzer, the position of which was determined using an argon ion cleaned Ag sample. No charging effect was observed during the photoelectron experiments. CasaXPS software was used to analyze XPS and UPS spectra. Gaussian and Lorentzian line shapes with Shirley-type backgrounds were applied. All measurements were made at room temperature.

## 3. Results

**Figure 1:**
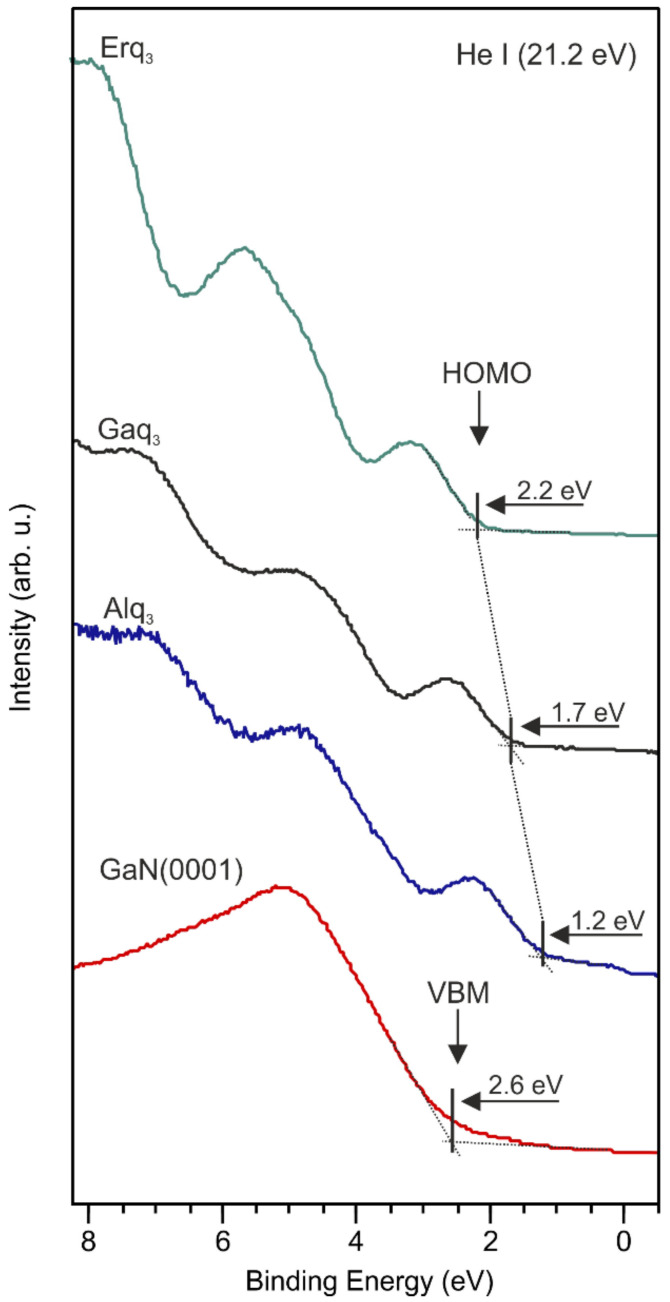
UPS spectra of valence band for bare p-GaN(0001) surface and covered with Alq_3_, Gaq_3_, Erq_3_ molecular layers ~7 nm thick. The same position of HOMO levels was for thicker layers.

Herein, the results are presented for 7 nm thick organic films, where electronic states of molecules are stabilized and are not affected by phase boundary electron transfer effects. Therefore, the position of the states is stabilized and does not depend on further thicknesses. The valence band of bare GaN(0001) surface and covered with Alq_3_, Gaq_3_, Erq_3_ is presented in Figure 1. The spectrum of the bare substrate reveals the valence band maximum (VBM) located at 2.6 eV below the *E*_F_. Separate deposition of Alq_3_, Gaq_3_, Erq_3_ molecules onto the bare GaN(0001) surfaces changes the shape of the valence band. When the surface is completely covered with the molecules, the appearance of an additional peak in the vicinity of the Fermi level is clearly visible. These electron states are recognized as the highest occupied molecular orbitals (HOMOs), their onsets are located at 1.2, 1.7, and 2.2 eV below the *E*_F_, respectively, for Alq_3_, Gaq_3_, and Erq_3_ layers. In the case of Alq_3_ molecules, the HOMO level is located in the same position for various coverages. The Gaq_3_ HOMO is clearly visible at the lowest coverage at 1.6 eV and shifts by 0.5 eV towards a higher binding energy with increasing film thickness and remains constant for coverages ≥7 nm, while the HOMO of Erq_3_ molecules behaves similar to Alq_3_. The positions were determined from the intersection of an extrapolated line fitted to the leading edge of the spectrum and its background. In the photoelectron energy distribution curves, other characteristic features are also visible. The maxima are recognized as deeper electron states of the molecules, i.e., HOMO-1, HOMO-2. The vacuum level (*E*_VAC_) of the bare substrate was located 4.3 eV above the *E*_F_, calculated from the equation *E*_VAC_ = *hv* − *E*_cutoff_, where *hv* = 21.2 eV is photon energy of He I line and *E*_cutoff_ is a cut-off of UPS spectrum.

**Figure 2:**
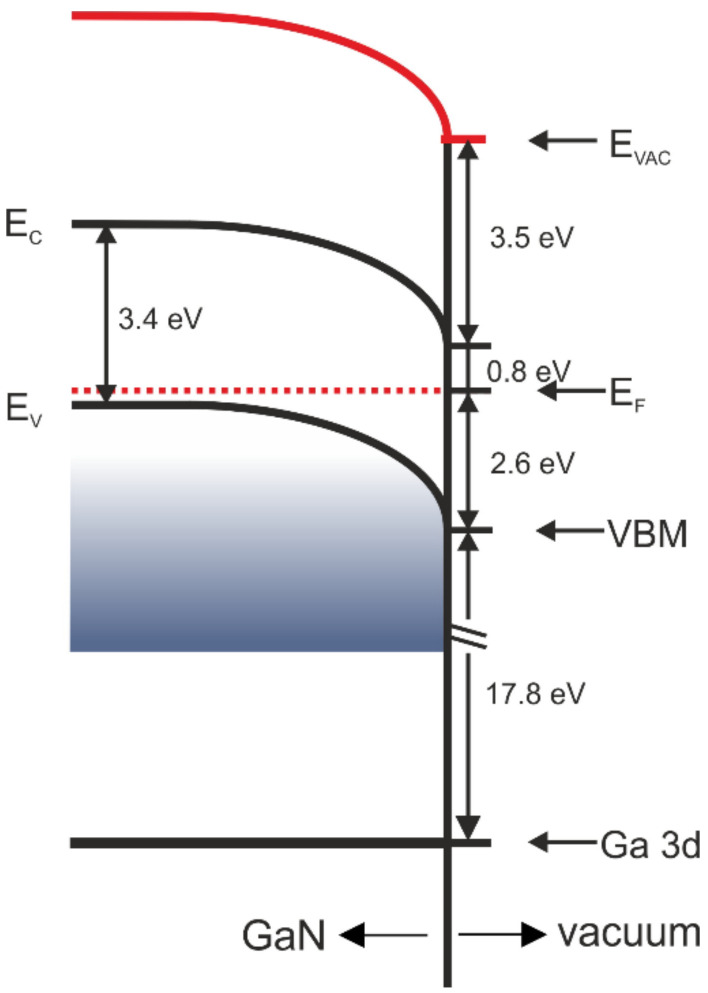
Energy band diagram for p-GaN(0001) substrate.

An electron affinity can be calculated from the equation $\chi =E_{\mathrm{VAC}}\;-\left(E_{g}-E_{\mathrm{VBM}}\right)$, where *E*_g_ is a band gap width and *E_VBM_* is a position of VBM. For the substrate, the electron affinity equals 4.3 eV. The UPS data allow constructing a sketch of energy bands for the initial GaN(0001) surfaces used in this experiment, as shown in Figure 2. The band bending of the bare substrates, induced by the Fermi level pinning at surface states, is in evidence. Assuming that the bulk Fermi level of the substrate is located 0.1 eV above the valence band maximum, the band banding is equal to 2.5 eV. Even though the substrates are p-type, the surface Fermi level is closer to the conduction band minimum than to the valence band maximum.

This result is in contrast to that in Refs. [30,31], which is most likely due to the fact that the initial surface of the substrates used in this report is depleted of holes. On GaN(0001) close to the conduction band minimum, there is a surface state which derives from Ga dangling bonds [32,33], thus, in the case of p-type GaN, the Fermi level pinning to this state leads to a strong band banding, which is the common observation [34,35,36,37]. Giving that the substrate is Mg-doped, the formation of depletion region is shown. The magnitude of band bending at the substrate needs to be included when trying to analyze the current–voltage characteristics of the device based on the inorganic-organic interface. As is shown further in the text, the magnitude can be changed after the phase boundary formation.

**Figure 3:**
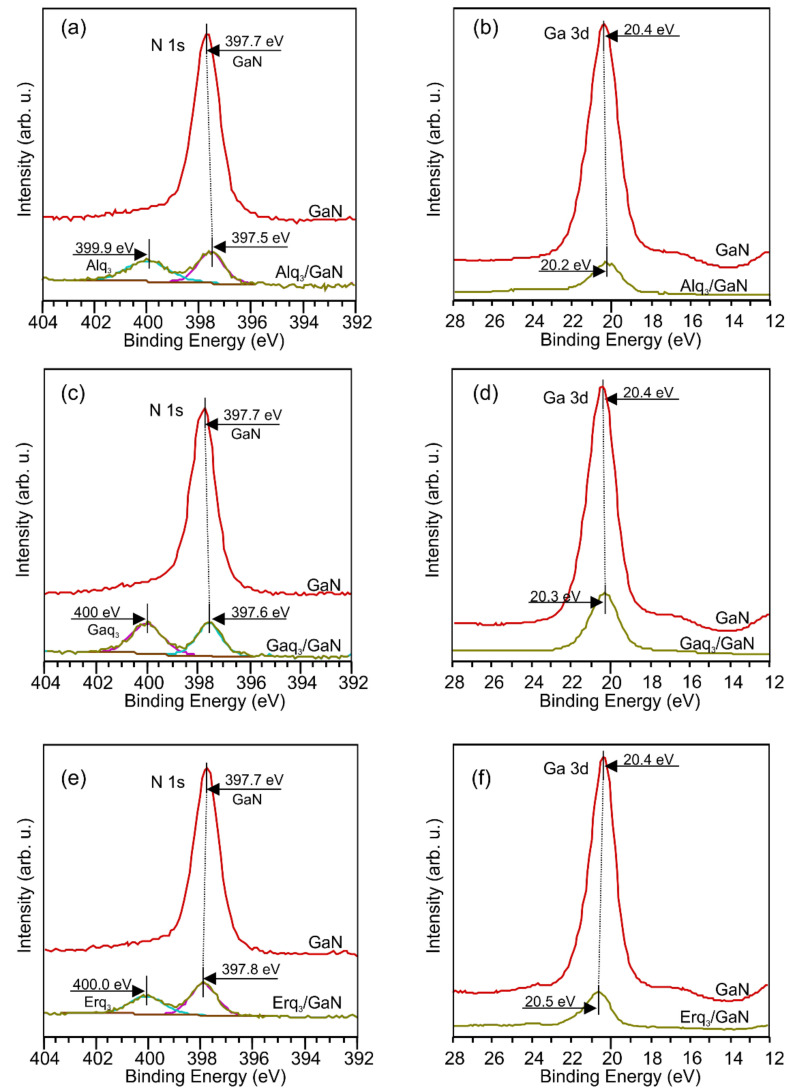
XPS spectra of the N1s and Ga 3d for bare p-GaN(0001) surface and covered with molecular layers ~7 nm thick. (**a**,**b**) Alq_3_, (**c**,**d**) Gaq_3_, and (**e**,**f**) Erq_3_ layers.

Different termination of the substrate surface generally leads to a vacuum level change. It is not the case for the GaN covered with Alq_3_ layer where the *E*_VAC_ does not alter, thus the work function change relative to the bare GaN(0001) equals zero (Δϕ = 0). The same vacuum level was observed for various Alq_3_ coverages. For Gaq_3_ the vacuum level systematically decreases with increasing film thickness. Finally, for coverages ≥7 nm it is located 3.5 eV above the *E*_F_, giving the work function change Δϕ = −0.8 eV. The highest change of the *E*_VAC_ was noted after deposition of Erq_3_, for which vacuum level was located 3.0 eV above the *E*_F_, giving Δϕ = −1.3 eV. The Erq_3_ vacuum level decreases with film thickness, similar to Gaq_3_ molecules. However, in order to reproduce the true change of work function Δϕ_D_ at the resulting inorganic-organic phase boundary, i.e., to determine the interface dipole, it is necessary to know whether there is an electron transfer at Mq_3_/GaN interface or not. When charging of the interface states occurs, the band bending of the substrate changes. The change leads to a shift of the *E*_VAC_ level for the substrate covered with molecules, the shift magnitude should be the same as the magnitude of the band bending change. To determine whether an electron transfer has occurred at the interface, it must be specified if the VBM position of the substrate has changed. Unfortunately, when the GaN(0001) surface is covered with an Mq_3_ layer, an additional density of states resulting from an overlapping of the ad-molecules’ orbitals with the GaN valence band prevents direct determination of the VBM from UPS measurements. Nevertheless, this measurement can be done indirectly using XPS. The core level lines for the substrate covered with the molecules are still visible since the mean free path for electrons from them is longer than for the valence band electrons. The positions of the VBM of the substrate for the three interfaces can be estimated based on the core level lines of the GaN substrate displacements, e.g., the Ga 3d or N 1s core level lines, after the molecule depositions considering the fact that the positions of the peaks relative to the VBM remain constant after ad-layer deposition. It is due to the fact the XPS results do not show indications of meaningful chemical interaction between the substrate and the adsorbed molecules. Figure 3 shows changes in the Ga 3d and N 1s peak positions caused by the presence of Alq_3_, Gaq_3_, Erq_3_ layers. One can see that the shifts of the peaks are the same, even in the case of Gaq_3_ molecules, where the Ga 3d state is derived from two sources (the substrate and the adsorbate). So the Ga 3d peak for the bare GaN(0001) lies 20.4 eV above the *E*_F_ and is located 17.8 eV above the VBM (see Figure 2). The latter value is constant and in line with other data [38,39,40].

Changes in the VBM positions relative to the value obtained for the bare surface indicate band bending modifications Δϕ_BB_ of the substrate. For the surface covered with an Alq_3_ layer, the band bending change gives Δϕ_BB_ = 0.2 eV. Smaller changes are observed for other molecules and so for Gaq_3_ Δϕ_BB_ = 0.1 and for Erq_3_ Δϕ_BB_ = −0.1 eV.

Knowing the changes of the work function Δϕ and band bending Δϕ_BB_, we can express the interface dipole as their sum, i.e., Δϕ_D_ = Δϕ + Δϕ_BB_. The values of interfacial polarization for the three Mq_3_/GaN systems are presented in Table 1.

**Figure 4:**
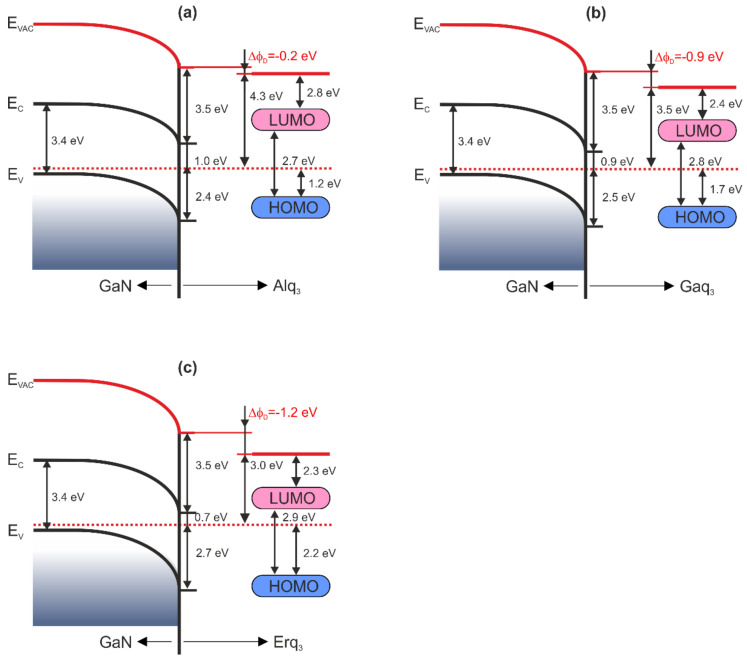
Energy level diagrams for Mq_3_/p-GaN(0001) interfaces. (**a**) Alq_3_, (**b**) Gaq_3_, and (**c**) Erq_3_.

The electron affinities for organic layers are 2.8, 2.4, 2.3 eV, respectively, for the Alq_3_, Gaq_3_, Erq_3_ molecules (assuming their band gaps are 2.7, 2.8, 2.9 eV [41,42,43]). The data allow constructing band diagrams for the three Mq_3_/GaN interfaces, as shown in Figure 4. The interfacial polarization Δϕ_D_ has the smallest value for the Alq_3_ film and the largest for the Erq_3_ film. This means that the higher is the number of electron shells of the central metal ion in the organic molecule Mq_3_, the higher is Δϕ_D_ (in absolute value).

When the Alq_3_ and Gaq_3_ molecules reduce the band bending of the inorganic substrate, the Erq_3_ molecules slightly increase it (relative to the bare substrate surface). The presence of organic molecules on the GaN(0001) nominally enables the work function reduction up to 1.2 eV. Knowing the electron affinity of the GaN substrate and the adsorbers as well as the interface dipole Δϕ_D_ of the systems unoccupied band offsets at the organic-inorganic interface can be expressed as:

$${\Delta E}_{C}=\chi_{s}-\chi_{o}+\Delta \phi_{D}.$$

To calculate the occupied band offsets, the band gaps of the semiconductors need to be included

$${\Delta E}_{V}=(\chi_{s}+E_{g})-(\chi_{o}+E_{\mathrm{go}})+\Delta \phi_{D}.$$

The band offsets between the conduction band and LUMO of the molecules are 0.5, 0.2, and 0 eV, respectively, for Alq_3_, Gaq_3_, Erq_3_ layers on GaN. Obtained data in this research are summarized in Table 2. The band offsets between the valence band and HOMO levels of the molecules Mq_3_ are 1.2, 0.8, 0.5 eV, for M = Al, Ga, Er, respectively. It means that that the higher is the number of electron shells of the central metal ion in the organic molecule Mq_3_, the lower is the distance between occupied bands of the inorganic and organic semiconductors. The above analysis shows that tuning of the vacuum levels, HOMO levels, and band offsets at the interfaces is possible by changing the central M atom of the molecule Mq_3_.

## 4. Conclusions

UPS assisted by XPS was used to investigate the electronic properties of the three Mq_3_/GaN(0001) interfaces. The electron affinity of the clean GaN(0001) surface was found to be 3.5 eV and the VBM position was measured to be 2.6 eV below the *E*_F_. HOMO levels were determined to be at 1.2, 1.7, 2.2 eV for the Alq_3_, Gaq_3_, Erq_3_ layers. The interface dipoles at the phase boundaries were amounted to be −0.2, −0.9, and −1.2 eV. The band offsets between the VBM of GaN(0001) and the HOMO level of the Alq_3_, Gaq_3_, Erq_3_ molecules amounted to 1.2, 0.8, and 0.5 eV. The research shows that the change of the central atom M in Mq_3_ molecules strongly impacts the electronic properties of the Mq_3_/GaN phase boundary.

## Figures and Tables

**Table 1:** Magnitudes of interface dipole for Mq_3_ on p-GaN(0001).

Organic Layer	Δϕ (eV)	Δϕ_BB_ (eV)	Δϕ_D_ (eV)
Alq_3_	0	−0.2	−0.2
Gaq_3_	−0.8	−0.1	−0.9
Erq_3_	−1.3	0.1	−1.2

**Table 2:** HOMO levels and band offsets for Mq_3_ on p-GaN(0001).

Organic Layer	HOMO (eV)	ΔC_V_ (eV)	ΔE_V_ (eV)
Alq_3_	1.2	0.5	1.2
Gaq_3_	1.7	0.2	0.8
Erq_3_	2.2	0	0.5

## Data Availability

The data presented in this study are available on request from the corresponding author.
